# In Vitro Investigation of Equine Gut Microbiota Alterations During Hypoglycin A Exposure

**DOI:** 10.3390/ani15223343

**Published:** 2025-11-19

**Authors:** Anne-Christine François, Bernard Taminiau, Benoît Renaud, Irma Elizabeth Gonza-Quito, Claire Massey, Carolyn Hyde, Richard J. Piercy, Caroline Douny, Marie-Louise Scippo, Georges Daube, Pascal Gustin, Véronique Delcenserie, Dominique-Marie Votion

**Affiliations:** 1 Department of Functional Sciences, Faculty of Veterinary Medicine, Pharmacology and Toxicology, Fundamental and Applied Research for Animals & Health (FARAH), University of Liège, 4000 Liège, Belgium; benoit.renaud@uliege.be (B.R.); p.gustin@uliege.be (P.G.); dominique.votion@uliege.be (D.-M.V.); 2 Department of Food Sciences—Microbiology, Faculty of Veterinary Medicine, Fundamental and Applied Research for Animals & Health (FARAH), University of Liège, 4000 Liège, Belgium; bernard.taminiau@uliege.be (B.T.); georges.daube@uliege.be (G.D.); 3 Laboratory of Food Quality Management, Department of Food Sciences, Faculty of Veterinary Medicine, Fundamental and Applied Research for Animals & Health (FARAH)—Veterinary Public Health, University of Liège, 4000 Liège, Belgium; iegonza@uliege.be (I.E.G.-Q.); veronique.delcenserie@uliege.be (V.D.); 4 Comparative Neuromuscular Diseases Laboratory, Royal Veterinary College, London NW1 0TU, UK; cmassey@rvc.ac.uk (C.M.); rpiercy@rvc.ac.uk (R.J.P.); 5 Bio-Analysis Centre, London NW1 0NH, UK; cali@b-ac.co.uk; 6 Laboratory of Food Analysis, Department of Food Sciences, Faculty of Veterinary Medicine, Fundamental and Applied Research for Animals & Health (FARAH), University of Liège, 4000 Liège, Belgium; cdouny@uliege.be (C.D.); mlscippo@uliege.be (M.-L.S.)

**Keywords:** equine atypical myopathy, microbiota, intestinal microbiota, horses, hypoglycin A, methylenecyclopropylacetyl-carnitine, 16S rRNA gene sequencing, next generation sequencing, toxin, poisoning, in vitro batch fermentation, short chain fatty acids

## Abstract

Hypoglycin A is a plant-derived protoxin that causes atypical myopathy in equids. In atypical myopathy-affected horses, metabolomic and microbiome studies have reported alterations in metabolic markers and faecal microbiota composition, pointing to a potential disruption of microbial homeostasis. However, in vivo observations are strongly confounded by host-related factors, underscoring the need for controlled in vitro approaches. To address this, we used an in vitro static batch fermentation model simulating the equine colon to investigate the direct effects of hypoglycin A on microbiota composition and activity. Faecal inocula from healthy horses were incubated in control and hypoglycin A-treated fermenters for 48 h, with serial analyses of hypoglycin A concentration, short-chain fatty acids, and 16S rRNA gene profiles. Hypoglycin A remained stable in the nutritive medium in the absence of microbiota, confirming that its degradation in inoculated fermenters was microbiota-dependent. The results showed significant microbial-associated hypoglycin A degradation without evidence of toxic metabolite formation. The analysis of α- and β-diversity revealed both an effect of incubation time, reflecting the natural temporal dynamics of microbial communities under batch fermentation, and a specific impact of hypoglycin A exposure, with certain taxa such as *Paraclostridium* being affected. This study provides the first in vitro evidence that the equine microbiota contributes to hypoglycin A degradation.

## Simple Summary

Atypical myopathy is a severe and often fatal poisoning of equids caused by the ingestion of sycamore maple tree seeds or seedlings that contain hypoglycin A. Once ingested, the protoxin is converted into harmful compounds that block energy production in muscles, leading to muscle breakdown. Scientists have questioned whether the microbes living in the gut could influence how this toxin behaves. Previous studies have shown differences in gut microbial communities between horses affected by atypical myopathy, their clinically healthy co-grazers, and a group of toxin-free horses serving as a control. This suggests that the microbiota may influence the outcome of intoxication. In this in vitro study, we recreated part of the horse’s large intestine in the laboratory and exposed it to hypoglycin A. Our results show that the toxin’s concentration decreased significantly when microbes were present, while no toxic breakdown products were detected. This study suggests that the equine gut microbiota may contribute to protection against hypoglycin A, providing new insights into the understanding and potential prevention of atypical myopathy.

## 1. Introduction

Atypical myopathy (AM) is a commonly fatal pasture-associated intoxication of equids that occurs predominantly during the autumn and spring in temperate European regions. The disease is strongly linked to the ingestion of seeds or seedlings of the sycamore maple (*Acer pseudoplatanus*), which contain plant-derived protoxins [1]. The analysis of plant materials from the sycamore maple tree showed that seedlings [2] and seeds [3] contain hypoglycin A (HGA) as well as methylenecyclopropylglycine (MCPrG) [4,5]. Both are non-proteinogenic amino acids structurally related to branched-chain amino acids (BCAAs), which likely allows them to enter the same metabolic pathways [6,7].

Although both HGA and MCPrG are present, HGA is considerably more abundant in seeds [4,8]. Moreover, HGA intoxication has been reported in humans and other animal species and is therefore better characterised in the literature [9,10,11,12,13,14,15,16,17]. Hypoglycin A is not toxic per se [18] but is metabolised into the toxic compound methylenecyclopropylacetyl-coenzyme A (MCPA-CoA) [19], which is responsible for the pathogenesis. The diagnosis of AM relies on the detection of its conjugated metabolites (i.e., methylenecyclopropylacetic acid-carnitine (MCPA-carnitine) and -glycine (MCPA-glycine)) and marked alterations of the acylcarnitines profile in serum and urine of AM horses [17,20,21,22,23], resulting from the inhibition of *β*-oxidation of fatty acids [24,25] and the inhibition of isovaleryl-CoA-dehydrogenase [21,24,25].

The gut microbiota constitutes a complex microbial ecosystem capable of metabolising a wide range of substrates, including plant secondary metabolites and toxins [26,27,28]. Depending on the enzymatic pathways involved, microbial activity can lead either to detoxification or to bioactivation of xenobiotics. Given its structure, HGA could be susceptible to microbial transformation or degradation, potentially influencing its toxicity.

The potential involvement of the microbiota in HGA poisoning has been suggested on several occasions in animals. One hypothesis proposes that ruminal transformation of HGA may occur, based on the observation that herbivorous species possessing a proximal fermentation chamber seem less susceptible to sycamore poisoning [11]. Supporting this hypothesis, an in vitro study using sheep ruminal fluid reported a decrease in HGA concentrations after incubation with either pure HGA or sycamore maple seeds. However, this effect was also observed, though to a lesser extent, in autoclaved ruminal fluid, suggesting that abiotic processes could contribute to the effect. Although the microbial markers used to evaluate community activity did not reveal significant differences [29], these markers may not provide sufficient resolution to accurately characterise microbiota dynamics [30].

In horses, which are hindgut fermenters, differences in faecal microbiota composition have been reported between AM-affected animals and healthy co-grazers [31,32]. In particular, an in vivo study comparing the faecal microbiota of AM-affected horses, co-grazers, and protoxin-free control horses revealed significant differences in *α*-diversity, evenness, and *β*-diversity, as well as in the relative abundance of several bacterial genera [32]. Metabolomic analyses further suggest the disruption of microbial homeostasis in the diseased animals [21]. Together, these findings indicate a possible interaction between HGA toxicity and gut microbial composition. Nonetheless, these in vivo studies are observational and subject to significant confounding by host factors such as diet, clinical condition, and environment [33,34].

Therefore, controlled in vitro approaches are essential to dissociate the host and microbial contributions. Batch fermentation models using equine faecal inocula are a well-established method to investigate microbial activity under defined conditions [35,36]. Nevertheless, no previous study has directly evaluated whether the equine hindgut microbiota can metabolise HGA or how exposure affects the microbial community structure. This represents the main gap addressed by the present work.

Based on existing evidence, we hypothesised that HGA toxicity may be influenced by the equine gut microbiota, which could transform or degrade the compound and, thereby, alter its bioavailability and effects. Specifically, we postulated that faecal microbial communities from healthy horses would be capable of transforming HGA in vitro, resulting in measurable decreases in protoxin concentration without the production of known toxic metabolites, and that HGA exposure would be associated with specific changes in microbial diversity, potentially reflecting some of the trends observed in vivo in horses exposed to HGA.

Using a static batch fermentation system, the objectives of this study were (i) to investigate, under in vitro conditions free from host influence, the modifications in colonic bacterial populations induced by HGA exposure compared with unexposed controls, and (ii) to characterise the temporal dynamics of HGA concentration within the batch fermentation system.

## 2. Materials and Methods

### 2.1. Selection of Donor Horses and Sampling

The inclusion criteria were as follows: (i) clinically healthy horses; (ii) normal general examination parameters; (iii) access to pasture for at least 6 h a day; and (iv) absence of detectable levels of HGA (aTRAQ^®^ kit (AB Sciex Germany GmbH, Darmstadt, Germany —LOQ 0.090 μmol/L [37]) and MCPA-carnitine (UPLC-MS/MS—LOD 0.001 nmol/L [38]) in blood serum. Sampling occurred in spring 2022.

All procedures in this study adhered to both national and international guidelines on animal welfare. The Animal Ethics Committee of the University of Liège had confirmed that the sampling process was part of routine veterinary practice to establish a diagnosis. As a result, formal ethical approval was not required. Informed consent was obtained from horse owners prior to the inclusion of the horses in the study.

The faecal samples were collected directly from the rectum in anaerobic jars and anaerobiosis was ensured using AnaeroGen^TM^ bags (Oxoid, Basingstoke, UK). The samples were transported under cooled conditions and stored at −80 °C until batch analysis. The pool of faeces was prepared by mixing equal proportions of samples from the two donors [39]. A phosphate-buffered solution containing per litre: 8.8 g of K_2_HPO_4_, 6.8 g of KH_2_PO_4_, and 0.1 g of sodium thioglycolate was used to prepare a 20% *w*/*v* faecal homogenate. Mechanical homogenisation was achieved in a Stomacher VWR^®^ Star-Blender LB400 (VWR International GmbH, Darmstadt, Germany). Double-coated sterile stomacher bags (300 × 190 mm) were used to collect and filter the faecal suspensions.

### 2.2. Static In Vitro Batch Model Derived from SHIME^®^ System

#### 2.2.1. Chemical Reagents and Consumable Materials

The materials and nutritional media were obtained from ProDigest (Ghent, Belgium). The nutritional medium, referred to as “feed”, used for the simulation of the gastrointestinal environment contained per litre of distilled water: arabinogalactan (1.2 g), pectin (2.0 g), xylan (0.5 g), glucose (0.4 g), yeast extract (3.0 g), special peptone (1.0 g), mucin (3.0 g), L-cystein-HCl (0.5 g), and starch (4.0 g). The prepared feed was autoclaved at 121 °C for 30 min.

#### 2.2.2. Determination of HGA Concentration in Batch System

An estimated maximum tolerated dose (MTD) of HGA in horses was determined thanks to a human equivalent dose (HED) of the MTD values for HGA in rats. The MTD of HGA in rats was determined with controlled diets over a 30-day period, and the result was 1.50 ± 0.07 mg HGA/kg BW/day [40].

The human equivalent dose (HED) was determined as previously described with the following mathematical formula [41] using K_m_ as a correction factor:

HED (mg/kg) = Animal dose (mg/kg) × (Animal K_m_/Human K_m_)

Then, the HED obtained was used to calculate the equivalent dose for horses using the same formula. 

The K_m_ of the horse was calculated based on its average height and weight (1.61 ± 0.073 m and 565.08 ± 69.81 kg, respectively) as previously described [42].

The Km was calculated by dividing the average body weight (BW, kg) of species by its body surface area (BSA, m^2^) as follows [41]:

K_m_ = BW (kg)/BSA (m^2^)

The BSA calculation is based on height and weight with the following mathematical formula [43]:

BSA (m^2^) = √ (Height (cm) × Weight (kg)/3600)

Consequently, the estimated MTD of HGA in horses was 0.08 mg HGA/kg BW/day or 45.4 mg HGA/day with the same average weight used (i.e., 565.08 ± 69.81 kg) [42]. The digestive volume of the gastrointestinal tract of an adult horse is around 200 L [44]: the concentration of HGA representing the MTD of HGA in the horse’s digestive system is 227 ng HGA/mL. Considering the acute exposure context of our in vitro fermentation system (i.e., 48 h), a 2-fold increase in HGA concentration (i.e., 454 ng HGA/mL) was tested. This concentration of HGA (purity 85%, Toronto Research Chemicals, Canada) was used in the three HTFs of the batch system.

#### 2.2.3. Static Batch Model and Sampling Procedure

The batch model is a short-term in vitro fermentation that uses the SHIME^®^ model to mimic the descending colon of the human intestinal tract [45,46]. For this study, the system was adapted to represent the descending colon in horses, maintaining a constant temperature of 38 °C and a pH range of 6.6–6.9 [44,47]. Indeed, the colon is the part of the digestive tract where most fermentation occurs in horses [47] and humans [48]. It is therefore the most appropriate environment in which to study the changes in the microbiota linked with the compounds ingested during feeding. Specifically, six double-jacketed fermenters were filled with 300 mL of feed and inoculated with the faecal homogenate (5% *v*/*v*). The fermenters were connected to a water bath that kept the system at a constant temperature. The pH was automatically controlled by adding either acid (0.5 M HCl) or base (0.5 M NaOH) as required to maintain values between 6.6 and 6.9. The anaerobic conditions were guaranteed by a N_2_ flush. Three fermenters served as “control fermenters” (CFs) containing only the nutritional media and faecal inoculate, while the other three additionally received a single addition of HGA (454 µg/L) and acted as “HGA-treated fermenters” (HTFs). This configuration allowed for obtaining triplicate results for each studied group.

The samples were obtained from each fermenter every 2 h for the first 24 h of the experiment, and every 6 h thereafter, until reaching 48 h. Consequently, the sampling time points were T0, T2, T4, T6, T8, T10, T12, T14, T16, T18, T20, T22, T24, T30, T36, T42, and T48. The system was started, and 10 min later, HGA was added to the HTF. At this moment, an additional sampling time point (T0*) was included to monitor the system immediately after HGA addition.

**Figure A1:**
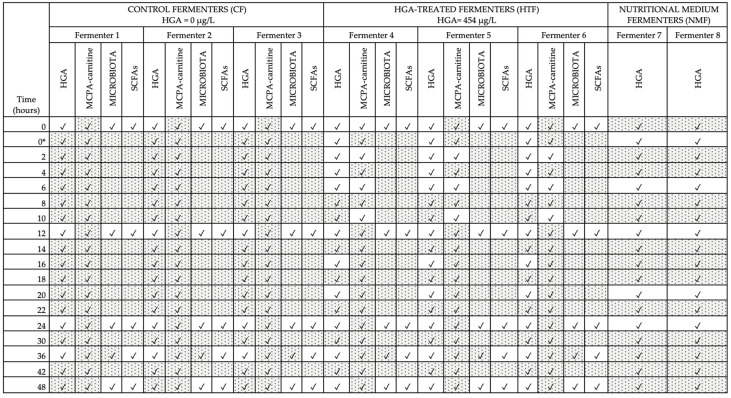
The first column indicates the sampling time. T0 corresponds to the baseline time point, while T0* refers to 10 min after adding HGA in HGA-treated fermenters. ✓ indicates the samples taken; white cells represent the samples that were analysed, whereas grey, textured cells represent the samples that were not analysed. HGA: hypoglycin A; MCPA-carnitine: methylenecyclopropylacetyl-carnitine; SCFAs: short-chain fatty acids.

Several samples were taken for different analyses, as shown in Figure A1, Appendix A: HGA and MCPA-carnitine quantification, short-chain fatty acids (SCFAs) quantification, and microbiota analysis. In practice, only a subset of samples was analysed to balance analytical depth and sample coverage.

### 2.3. Microbiota Assessment

#### 2.3.1. Bacterial DNA Extraction and High-Throughput Sequencing

The total bacterial DNA was extracted from faecal samples using the PSP Spin Stool DNA Plus Kit 00310 (Invitek, Berlin, Germany), following the manufacturer’s instructions. The PCR amplification of the 16S rDNA V1–V3 hypervariable region and the library preparation were performed using the following primers with Illumina overhang adapters: forward (5′-GAGAGTTTGATYMTGGCTCAG-3′) and reverse (5′-ACCOGCOGGCTGCTGGCAC-3′) [49].

The PCR products were purified using the Agencourt AMPure XP bead kit (Beckman Coulter, Pasadena, CA, USA), followed by a second PCR round for indexing with Nextera XT index primers 1 and 2. After purification, amplicons were quantified with Quant-IT PicoGreen (ThermoFisher Scientific, Waltham, MA, USA) and diluted to 10 ng/μL.

Final quantification was conducted using the KAPA SYBR^®^ FAST qPCR Kit (Kapa Biosystems, Wilmington, MA, USA) before normalisation, pooling, and sequencing on a MiSeq platform with V3 reagents (Illumina, San Diego, CA, USA). The sequencing run included commercial mock community positive controls containing DNA from 10 defined bacterial species (ATCC MSA-1000, ATCC, Manassas, VA, USA) as well as negative controls from the extraction and PCR steps [50].

Raw amplicon sequencing libraries were submitted to the NCBI database under bioproject number PRJNA1335877.

#### 2.3.2. Sequence Analysis and 16S rDNA Profiling

Sequence read processing was performed as previously described [50] using the Mothur software package v1.48 [51] and the VSEARCH algorithm for chimaera detection [52]. For operational taxonomic unit (OTU) generation, a clustering distance of 0.03 was used. Briefly, 16S reference alignment and taxonomical assignment, from phylum to genus, were performed with Mothur and were based upon the SILVA database (v138.1) of full-length 16S rDNA sequences [53]. These OTUs were further clustered into a final relative abundance table at the genus level.

#### 2.3.3. Microbiota Analysis

Subsampled datasets with 10,000 cleaned reads per sample were obtained and used to evaluate α-diversity (i.e., measuring diversity within the community) and β-diversity (i.e., measuring diversity between communities or within the same community at different time points by considering sequence abundances or by considering only the presence–absence of sequences) using the vegan R package (v 2.6-6.1) [54,55,56].

**Figure A2:**
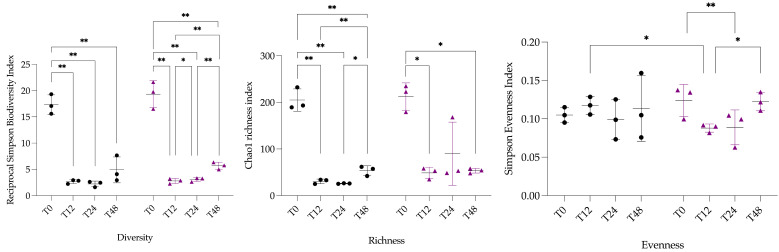
Representation of bacterial α-diversity derived from Reciprocal Simpson Biodiversity index, bacterial genus richness derived from Chao1 index, and bacterial genus evenness derived from Simpson Evenness index. Data are scatter dot plots at genus level for each fermenter in both groups (i.e., control fermenters and HGA-treated fermenters), with mean and standard deviation. Horizontal bars indicate the mean, and vertical bars indicate the standard deviation for each group. Black circles represent control fermenters, and purple triangles represent hypoglycin A-treated fermenters. Groups were compared with ANOVA followed by post hoc pairwise tests, using FDR multi-tests correction (*q*-value threshold: * <0.05; ** <0.01).

The indicators of *α*-diversity computed are the reciprocal Simpson microbial diversity index, Chao richness index (i.e., richness which quantifies the number of species present within a community), and Simpson-derived evenness index (i.e., evenness which describes how uniformly individuals are distributed among the species, highlighting the presence or dominance of certain species) [55,56,57]. Differences in *α*-diversity between groups (CF vs. HTF) were evaluated with an ANOVA test (with a Geisser–Greenhouse correction) followed by paired post hoc tests corrected for multiple comparisons by controlling the False Discovery Rate (FDR) with a two-stage linear step-up procedure of Benjamini, Krieger, and Yekutieli using PRISM 10 (GraphPad Software; San Diego, CA, USA), and were considered significant for a *p* and *q*-value of 0.05 or less. As T0 represents the moment when the system was started following pooling of donor faeces, the microbial populations inevitably have to adapt to this new environment (i.e., descending colon). Consequently, T0 is expected to be statistically different from subsequent time points. Moreover, no treatment (i.e., HGA addition) was applied at T0, and the two groups were therefore not different. As a result, the figure presented in the main text excludes T0 from the *α*-diversity statistical analysis, while a second informative figure including this time point is presented in Figure A2, Appendix B.

The *β*-diversity was analysed using vegan and vegan3d packages (v 1.3-0) in R [58]. A principal coordinate ordination analysis (PCoA) based on the Bray–Curtis dissimilarity matrix was used to visualise samples in *β*-diversity analysis. Differences between groups were assessed with a PERMANOVA (Adonis2) and post hoc pairwise comparisons (pairwise adonis package v 0.4.1) [59] with a significance threshold *p* of 0.05. These analyses were conducted on both the complete dataset and after T0 exclusion. A distance-based redundancy analysis (dbRDA) was further applied as a constrained ordination model to assess the effect of the variables *Time* and *Treatment* on the *β*-diversity. This step of the statistical analysis was also realised without the T0. The significance of the model and axes was assessed with ANOVA.

Finally, a differential abundance analysis was performed using the DESeq2 package in R (v1.44.0) to examine statistically significant changes in microbial abundance between treatments and across time points. This step was also carried out after excluding T0.

### 2.4. Quantification of Short Chain Fatty Acids and Statistical Analysis

A previously validated method of solid-phase microextraction (SPME), followed by gas chromatography coupled to mass spectrometry (GC–MS) [60], was used to simultaneously quantify the SCFAs produced in each fermenter at T0, T12, T24, T36, and T48. The measurement of SCFAs was essential, as they represent major metabolic end products of microbial fermentation and serve as indicators of intestinal microbiota activity and functionality within the batch model.

The analytical technique has the following lower and upper limits of quantification (LLOQ and ULOQ, respectively): acetic acid (C2): 2.00–99.90 mmol/L, propionic acid (C3): 0.97–48.60 mmol/L, butyric acid (C4): 0.57–28.37 mmol/L, isobutyric acid (iC4): 0.16–7.94 mmol/L, isovaleric acid (iC5): 0.10–4.90 mmol/L, valeric acid (C5): 0.29–14.69 mmol/L, caproic acid (C6): 0.0086–0.43 mmol/L, heptanoic acid (C7): 0.008–0.04 mmol/L, and octanoic acid (C8): 0.0007–0.03 mmol/L [60].

The proportion of each SCFA as a percentage of the total of SCFAs was calculated. The statistical analyses of SCFA concentrations were performed using GraphPad Prism 10 (GraphPad Software; San Diego, CA, USA) with a *p* threshold value of 0.05. The values which were under the LLOQ of the methods were replaced by the value of the LLOQ for the statistical analysis. The D’Agostino–Pearson, Anderson–Darling, Shapiro–Wilk, and Kolmogorov–Smirnov tests were used to assess the normality or lognormality of the data distribution. A two-way ANOVA was performed for each SCFA with a Greenhouse–Geisser correction, followed by post hoc multiple comparisons adjusted using Tukey’s method.

### 2.5. Quantification of Hypoglycin A and Statistical Analysis

Hypoglycin A concentrations were quantified using liquid chromatography coupled with mass spectrometry, without derivatisation, as previously described by González-Medina et al. (2021) [61]. The LOD of this method is 0.055 ng/mL.

Statistical analyses of HGA concentrations were conducted using GraphPad Prism 10 (GraphPad Software, San Diego, CA, USA), with a significance threshold set at *p* < 0.05.

#### 2.5.1. Preliminary Assessment of Hypoglycin A Stability in the Nutritional Medium

To evaluate possible degradation of HGA, two additional fermenters containing 300 mL of autoclaved nutritional medium were prepared, and HGA was added (i.e., 150 ng HGA/mL). The temperature (i.e., 38 °C) was maintained by a water bath. The pH was controlled before each sampling and adjusted manually if needed by the addition of 0.5 M NaOH or 0.5 M HCl. For this parallel experience, sampling points were: T0*, T6, T12, T20, and T24. These fermenters are designed as “nutritional medium fermenters” or NMFs.

Normality and lognormality tests (D’Agostino–Pearson, Anderson–Darling, Shapiro–Wilk, and Kolmogorov–Smirnov tests) were performed. An ANOVA with a Greenhouse–Geisser correction was performed, followed by a multiple comparisons test, which compared the mean concentration of HGA of each time point to the T0* considered as “the control time point” with a correction of False Discovery Rate (FDR) of Benjamini, Krieger, and Yekutieli (*q* = 0.05).

#### 2.5.2. Hypoglycin A Concentration in the Batch Fermenters: Data Analysis

Time points were selected to provide a representative overview of HGA degradation over the incubation period while maintaining a balance between analytical depth and the number of samples processed.

Changes in HGA concentration over time (T0, T12, T24, T36) were analysed within the CF. Normality and lognormality were assessed using the D’Agostino–Pearson, Anderson–Darling, Shapiro–Wilk, and Kolmogorov–Smirnov tests. Data were log-transformed when required to meet distribution assumptions. A repeated measures one-way ANOVA was then performed, applying the Greenhouse–Geisser correction. Multiple comparisons were first carried out using Dunnett’s test to compare each time point to the baseline (T0). When statistically significant differences were detected, additional pairwise comparisons were performed using Tukey’s correction to assess differences between all the time points.

The same statistical approach was subsequently applied to the HTF, analysing HGA concentrations at T0, T0*, T2, T4, T6, T12, T16, T20, T24, T36, and T48. However, because one of the three replicates at T6 was missing, the repeated measures one-way ANOVA for the HTF was replaced by a mixed-effects analysis. 

Finally, a two-way ANOVA was performed to compare both groups (CF vs. HTF) at the time points common to both groups (T0, T0*, T12, T24, and T36), and a multiple comparison test with Tukey’s correction was applied. The values at T0* for the CF were supposed to be the same as the values at T0, which made analysis between the two groups possible (CF vs. HTF).

#### 2.5.3. Kinetic Data Analysis of Hypoglycin A Concentration

In addition to the ANOVA tests performed to compare HGA concentrations between time points, linear regression analyses were conducted to compare the overall degradation trend within CFs, HTFs, and NMFs. The slope of the regression line was used as an indicator of the apparent degradation rate of HGA. The significance of the regression was assessed using the F-test for the null hypothesis that the slope equals zero.

### 2.6. Quantification of Methylenecyclopropylacetyl-Carnitine

An ultra-performance liquid chromatography combined with subsequent tandem mass spectrometry (UPLC-MS/MS) was used for MCPA-carnitine quantification, as previously described [38]. The LOD is 0.01 nmol/L. The MCPA-carnitine was quantified only in HTF.

## 3. Results

### 3.1. Donor Horses

Two horses were involved in the study.

Horse 1: The horse was an 8-year-old Zangersheide female with a 5/9 body-score. The horse lived at pasture for more than 6 h a day and received hay (12 kg/1 time a day). The horse had access to a salt block. The horse is a leisure horse and was ridden 3 times/week for 1 h session each time.

Horse 2: The horse was a 3-year-old Haflinger and Spanish cross-breed female with a 5/9 body-score. The horse lived at pasture for more than 6 h a day and received ad libitum hay. The horse had access to a salt block. The horse is a leisure horse.

### 3.2. Microbiota Analysis

Starting with 2,760,444 raw reads, 2,050,624 reads were kept after read cleaning and chimaera removal. We proceeded with 10,000 reads per sample to taxonomic identification, leading to a table of 3489 OTUs. The determination of *α*- and *β*-diversities of the bacterial populations were assessed at the genus level.

**Figure 1:**
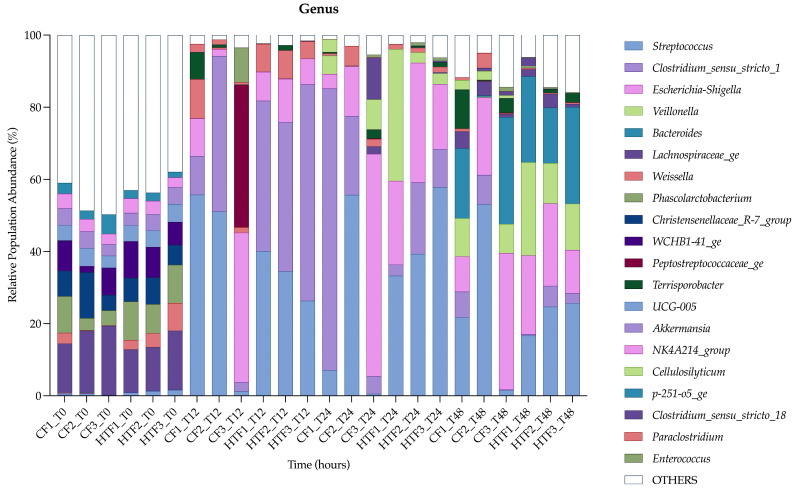
Main dominant genera at each time point for each control fermenters (CF) and hypoglycin A-treated fermenters (HTF) in relative population abundance by percentage.

The relative abundance of genera identified is represented in Figure 1. The genera at T0 seemed to be different from those at the other time points. The most represented genus at T0 was *Lachnospiraceae*_*ge*. The five most represented genera at T12, T24, and T48 were *Streptococcus*, *Clostridium_sensu_stricto_1*, *Escherichia-Shigella*, *Veillonella*, and *Bacteroides*. The decrease in the proportion of the “OTHERS” genera over time reflects the general dynamic of the batch system.

#### 3.2.1. α-Diversity Analysis

**Figure 2:**
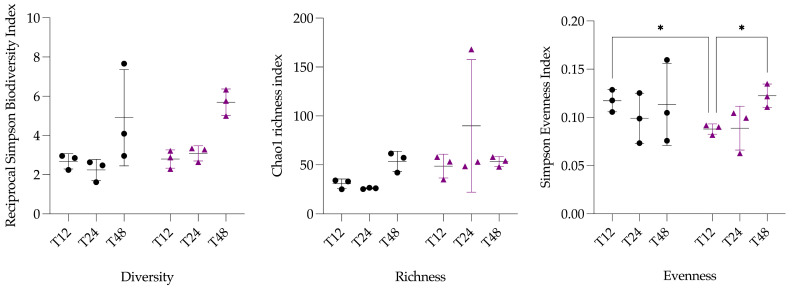
Representation of bacterial *α*-diversity derived from Reciprocal Simpson Biodiversity index, bacterial genus richness derived from Chao1 index, and bacterial genus evenness derived from Simpson Evenness index. Data are scatter dot plots at the genus level for each fermenter in both groups (i.e., control fermenters and HGA-treated fermenters). Horizontal bars indicate the mean, and vertical bars indicate the standard deviation for each group. Black circles represent control fermenters, and purple triangles represent HGA-treated fermenters. Groups were compared with ANOVA followed by post hoc pairwise tests, using FDR multiple-test correction (*q*-value threshold: * <0.05).

The *α*-diversity indexes (i.e., richness, diversity, and evenness) were compared between treatment groups (*Treatment* effect) and within groups (*Time* effect). Concerning the Reciprocal Simpson Index (i.e., diversity), a significant effect of *Time* was observed (*p* = 0.0320, *). The evenness (i.e., Simpson Evenness Index) was significantly different between CF vs. HTF at T12 (*q* = 0.0329, *) and between T12 vs. T48 (*q* = 0.0319, *) in the HTF (Figure 2).

#### 3.2.2. β-Diversity Analysis

**Figure 3:**
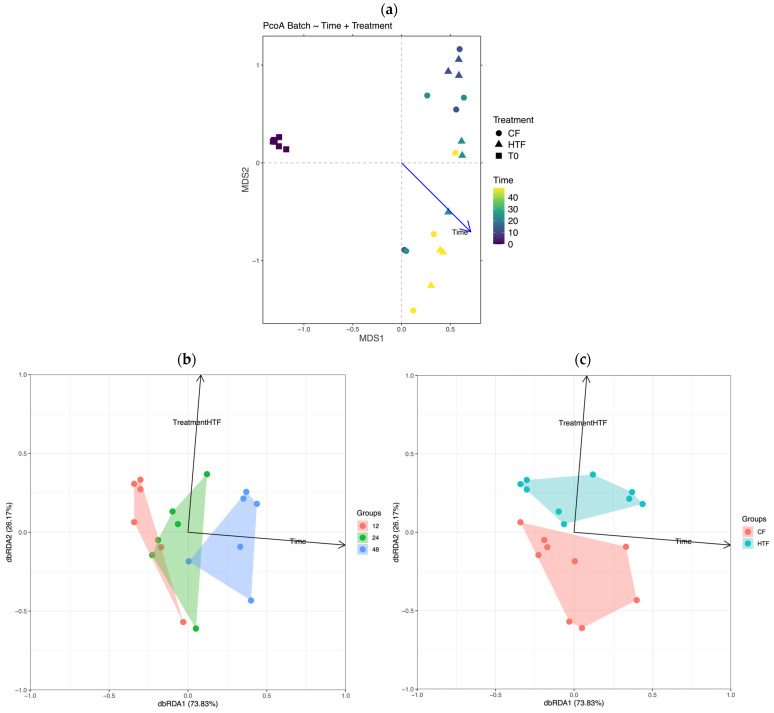
Representation of bacterial *β*-diversity analysis with (**a**) Principal Coordinates Analysis—Rounds symbols represent control fermenters (CF) and triangular symbols represent hypoglycin A-treated fermenters (HTF) after adding hypoglycin A, while square symbols represent fermenters at T0 before adding HGA, (**b**) Bray–Curtis-based Redundancy Analysis model of microbiota samples for T12, T24, and T48 coloured by *Time* value and (**c**) Bray–Curtis-based Redundancy Analysis model of microbiota samples coloured by *Treatment* group. Variable fitting to model is represented by a vector for each variable for both Bray–Curtis-based Redundancy Analyses.

The first PERMANOVA *β*-diversity analysis, including the interaction between *Time* and *Treatment*, revealed a significant effect of both factors and their interaction (*p* = 0.001, ***). However, inclusion of T0 samples influenced the ordination pattern (Figure 3a).

To better capture the effects of the experimental factors independently from baseline, a second PERMANOVA was performed after excluding T0 samples. This analysis confirmed a significant global effect (*p* = 0.014, *) explaining 33.6% of the total variance in microbial community structure. The factor *Time* alone accounted for 26.2% of the variance and had a significant impact on β-diversity (*p* = 0.001, ***), whereas *Treatment* explained only 6.6% of the variance and was not significant. A permutation design stratified by *Time* confirmed the absence of a *Treatment* effect (*p* = 0.264). Pairwise comparisons for the *Time* factor are summarised in Table 1. The influence of both *Time* and *Treatment* was further illustrated by a db-RDA constrained ordination model (Figure 3b,c), for which only the first axis (db-RDA1) and the *Time* factor were significant (*p* = 0.002, **).

#### 3.2.3. Differences in Microbiota Composition Between Groups

**Figure 4:**
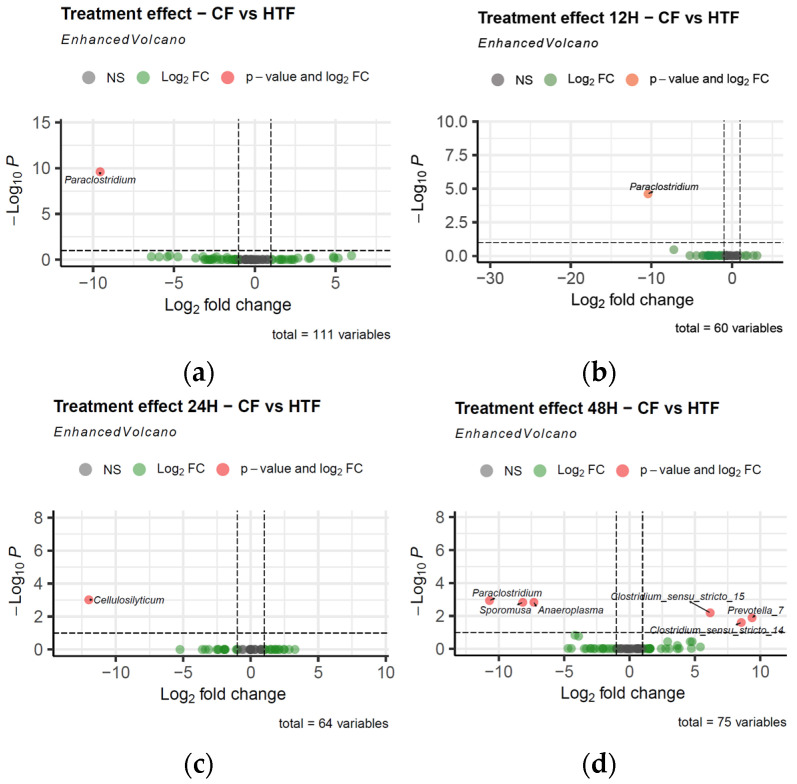
Volcano plot for *Treatment* effect with differences in microbial population abundance between control fermenters (CF) and hypoglycin A-treated fermenters (HTF). (**a**) all *Time* combined; (**b**) T12; (**c**) T24; (**d**) T48. X-axis represents log2 fold change, indicating relative change between two conditions: CF on left and HTF on right. Y-axis represents −log10 *p*-value. Horizontal dotted line indicates significant threshold (*p* = 0.05). Red dots represent populations that are significantly different between CF and HTF.

**Figure 5:**
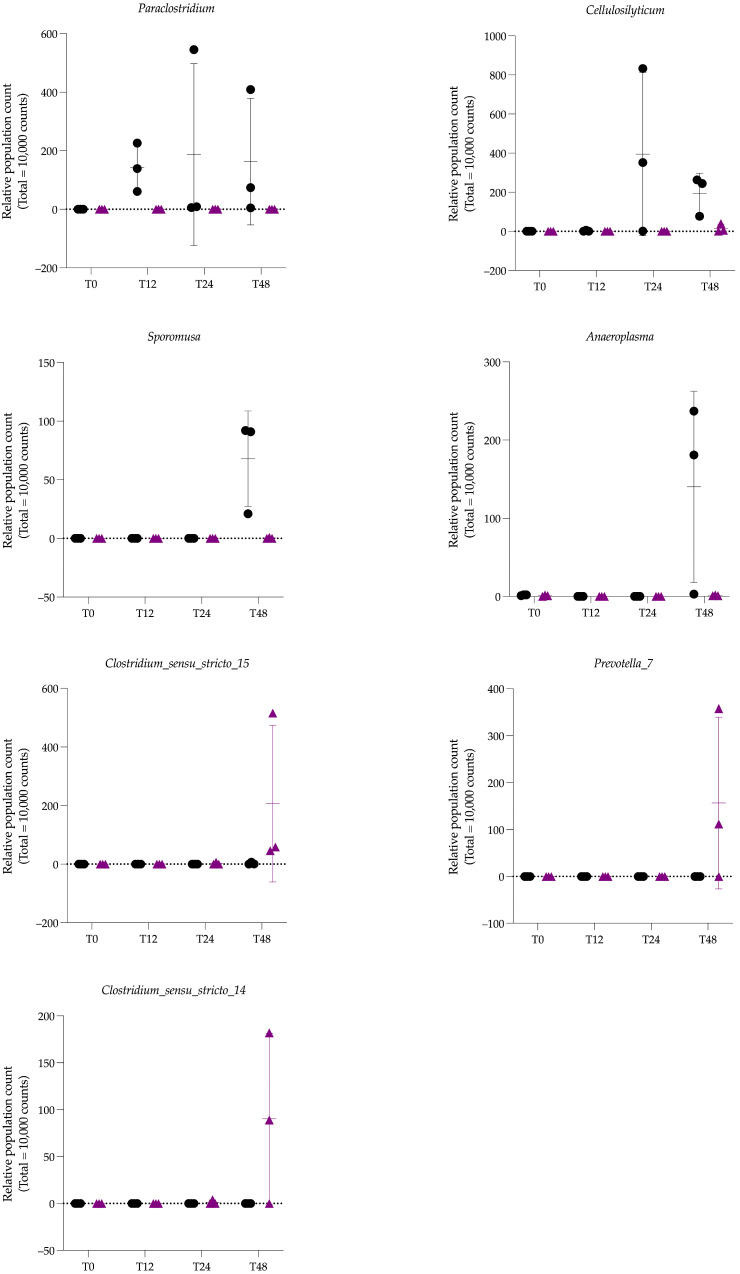
Graphical representation of relative population count (total 10,000 counts). Each symbol represents one fermenter: black circles correspond to control fermenters and purple diamonds correspond to hypoglycin A-treated fermenters. Horizontal bars indicate the mean, and vertical bars indicate the standard deviation for each group.

Differences in microbial population abundance between CF vs. HTF were assessed with Deseq2. The analysis revealed a significantly higher abundance of the *Paraclostridium* genus in the CF group compared to HTF (*p* = 2.5136 × 10^−10^, ***) (Figure 4). Pairwise comparisons between *Treatment* groups at each sampling time highlighted several genera, as shown in the volcano plots (Figure 5). At T12, the only significant genus between CF and HTF was *Paraclostidium* (*p* = 2.4113 × 10^−05^, ***). At T24, the significant genus between groups was *Cellulosilyticum* (*p* = 9.6744 × 10^−04^, ***). At T48, significantly different genera between groups included *Paraclostridium* (*p* = 1.1703 × 10^−03^, **), *Sporomusa* (*p* = 1.4903 × 10^−03^, **), *Anaeroplasma* (*p* = 1.4903 × 10^−03^, **), *Clostridium sensu stricto 15* (*p* = 6.4399 × 10^−03^, **), *Prevotella 7* (*p* = 1.2989 × 10^−02^, *), and *Clostridium sensu stricto 14* (*p* = 2.5008 × 10^−02^, *). The genera *Paraclostridium*, *Cellulosilyticum*, *Sporomusa*, and *Anaeroplasma* were mainly present in CF (i.e., in the left part of the volcano plots) while *Clostridium sensu stricto 15*, *Prevotella 7*, and *Clostridium sensu stricto 14* were mainly present in HTF (i.e., in the right part of the volcano plots). These differences are illustrated in Figure 5, which displays the relative abundance (population count) of each genus across time points.

### 3.3. Quantification of Short Chain Fatty Acids and Statistical Analysis

Calculated across all sampling times, the proportion of acetate, propionate, and butyrate was 85 ± 13.7%, 16 ± 5.8%, and 8 ± 2.1%, respectively, in CF and 79 ± 14.4%, 19 ± 9.4%, and 10 ± 4.1%, respectively, in HTF.

Two-way ANOVA analysis performed on SCFAs proportions pooled across all sampling times revealed a significant effect of “SCFAs” (*p* ≤ 0.0001, ****) and *Treatment* (*p* = 0.0207, *).

Two-way ANOVA analysis of acetate concentrations revealed a significant effect of *Time* (*p* = 0.0002, ***). Post hoc comparisons showed significant differences: (1) in the CF, between T0 and T36 (*p* = 0.0279, *) and between T0 and T48 (*p* = 0.0178, *); (2) in the HTF, between T0 and T12 (*p* = 0.0294, *), T0 and T24 (*p* = 0.0099, **), and T0 and T36 (*p* = 0.0241, *).

Two-way ANOVA analysis of propionate concentrations indicated a significant effect of *Time* (*p* = 0.0016, **). Post hoc comparisons revealed that significant pairwise differences occurred only in the HTF group, specifically between T0 and T36 (*p* = 0.0090, **), T0 and T48 (*p* = 0.0050, **), T12 and T36 (*p* = 0.0090, **), and between T12 and T48 (*p* = 0.0050, **).

**Figure 6:**
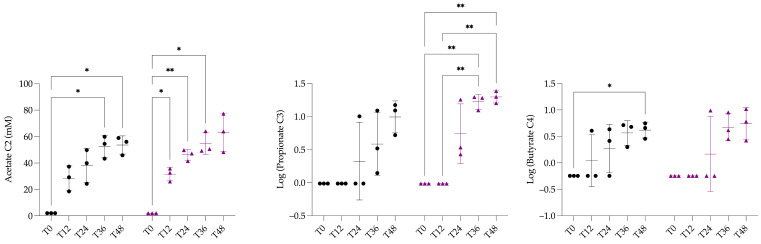
Graphical representation of acetate concentration (mM) and log-transformed concentration of propionate and butyrate in batch experiment. Black circles represent individual measurements from control fermenters, and purple diamonds represent individual measurements from hypoglycin A–treated fermenters. Horizontal bars indicate the mean, and vertical bars indicate the standard deviation for each group. Significantly different with a *p*-value of 0.05 or less: * <0.05; ** <0.01.

Two-way ANOVA analysis of butyrate concentrations presented a significant effect of *Time* (*p =* 0.0038, **) and *Fermenter* (*p* = 0.0241, *). Post hoc comparisons showed a significant difference in the CF between T0 and T48 (*p* = 0.0300, *). No difference was revealed between CF and HTF for any of the SCFAs analysed (Figure 6).

### 3.4. Quantification of Hypoglycin A and Statistical Analysis

#### 3.4.1. Stability of Hypoglycin A in the Nutritional Medium

No statistical difference was found at the sampling time points, indicating that HGA remains stable in the autoclaved nutritional medium when the pH and temperature are kept constant.

#### 3.4.2. Hypoglycin A Concentration in the Control Fermenters

**Figure 7:**
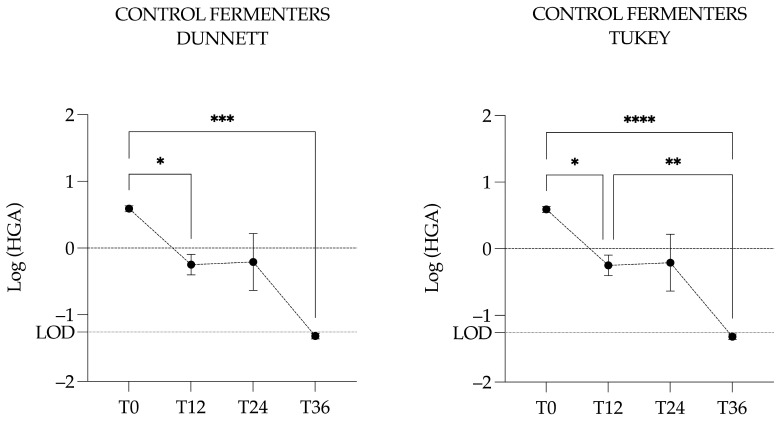
Graphical representation of the log-transformed concentration of hypoglycin A in the control fermenters. Circles represent the mean concentration across the three control fermenters, and vertical bars indicate the corresponding standard deviation. The LOD is the limit of detection and is expressed in Log (LOD). Significantly different with a *p*-value of 0.05 or less: * <0.05; ** <0.01; *** <0.001; **** <0.0001.

A repeated measures one-way ANOVA was performed with either the Dunnett or Tukey correction. The multiple comparison test with the Dunnett correction was applied to compare each time point to the control reference (i.e., T0) and revealed a significant difference between T0 and T12 (*p* = 0.0128, *) and between T0 and T36 (*p* = 0.0001, ***). The multiple comparison test with the Tukey correction was applied to identify specific time points that differed significantly from others. This second test revealed significant differences between T0 and T12 (*p* = 0.0167, *) and T36 (*p* ≤ 0.0001, ****) but also between T12 and T36 (*p* = 0.0095, **) (Figure 7).

#### 3.4.3. Hypoglycin A Concentration in the Hypoglycin A-Treated Fermenters

Considering T0 as the reference time, the multiple comparison test with the Dunnett’s correction revealed that T0 was significantly different from T0* (*p* = 0.0110, *), T2 (*p* = 0.0207, *), T4 (*p* = 0.0418, *), T12 (*p* = 0.0085, **), T16 (*p* = 0.0143, *), and T20 (*p* = 0.0196, *).

**Figure 8:**
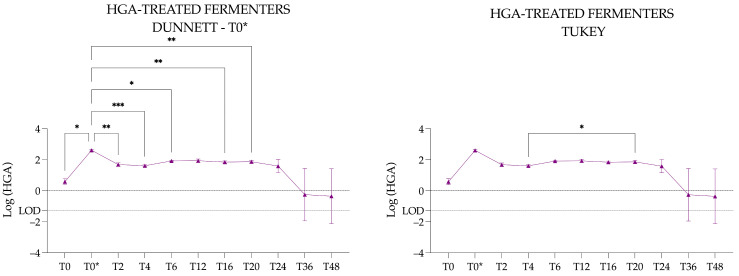
Graphical representation of the log-transformed concentration of hypoglycin A in the hypoglycin A-treated fermenters. Triangles represent the mean concentration across the three hypoglycin A-treated fermenters, and vertical bars indicate the corresponding standard deviation. The LOD is the limit of detection and is expressed in Log (LOD). Significantly different with a *p*-value of 0.05 or less: * <0.05; ** <0.01; *** <0.001.

Considering T0* (i.e., after adding HGA) as the reference time, the multiple comparison test with the Dunnett’s correction revealed that T0* was significantly different from T0 (*p* = 0.0110, *), T2 (*p* = 0.0087, **), T4 (*p* = 0.0002, ***), T6 (*p* = 0.0189, **), T16 (*p* = 0.0078, **), and T20 (*p* = 0.0043, **) (Figure 8).

The Tukey multiple comparison test identified significant differences between all the time points without using a reference time: T0 vs. T0* (*p* = 0.0176, *), T0 vs. T2 (*p* = 0.0341, *), T0 vs. T12 (*p* = 0.0142, *), T0 vs. T16 (*p* = 0.0232, *), T0 vs. T20 (*p* = 0.0322, *), T0* vs. T2 (*p* = 0.0145, *), T0* vs. T4 (*p* ≤ 0.0001, ****), T0* vs. T6 (*p* = 0.0036, **), T0* vs. T16 (*p* = 0.0134, *), T0* vs. T20 (*p* = 0.0089, **), and T4 vs. T20 (*p* = 0.0424, *). Tukey test results are presented to emphasise significant differences between time points that were not identified by the Dunnett test (Figure 8).

#### 3.4.4. Hypoglycin A Concentration Comparison Between Control Fermenters and Hypoglycin A-Treated Fermenters

The two-way ANOVA on log-transformed data revealed a significant effect of *Time* (*p* = <0.0091, **) and *Treatment* (*p* = 0.0056, **). Moreover, the source of variation is explained by *Time* for 40.38%, by *Treatment* for 33.85%, by the interaction of “*Time* × *Treatment*” for 10.92%, and, finally, by the factor *Fermenter* for 4.62%.

**Figure 9:**
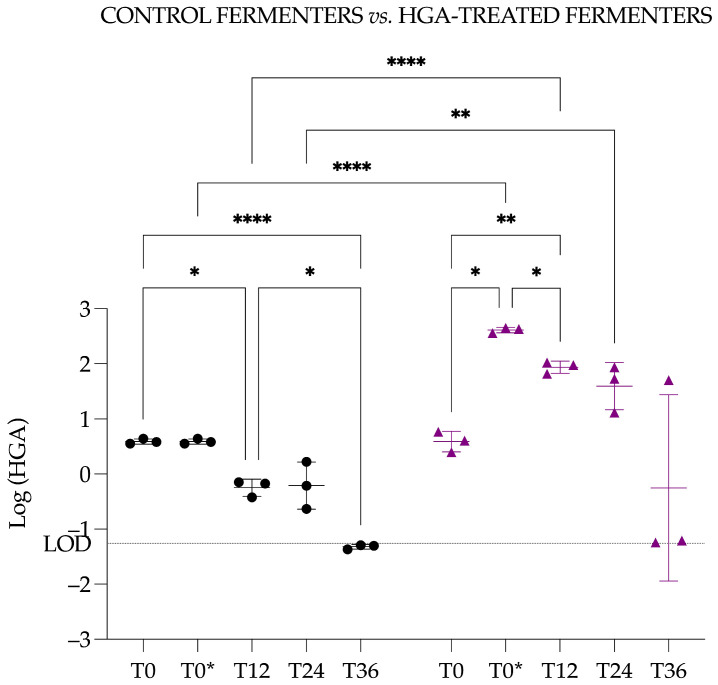
Graphical representation of the log-transformed concentration of hypoglycin A in the control fermenters and in the hypoglycin A-treated fermenters. Black circles represent individual measurements from control fermenters, and purple diamonds represent individual measurements from hypoglycin A–treated fermenters. Horizontal bars indicate the mean, and vertical bars indicate the standard deviation for each group. The LOD is the limit of detection and is expressed in Log (LOD). Significantly different with a *p*-value of 0.05 or less: * <0.05; ** <0.01; **** <0.0001.

Moreover, Tukey’s multiple comparison test revealed that (1) the HGA concentration was significatively different between CF and HTF at T0* (*p* ≤ 0.0001, ****), T12 (*p* ≤ 0.0001, ****), and T24 (*p* = 0.0067, **); (2) within the CF, the significant differences were T0 vs. T12 (*p* = 0.0204, *), T0 vs. T36 (*p* ≤ 0.0001, ****), and T12 vs. T36 (*p* = 0.0117, *); (3) within the HTF, the significant differences were T0 vs. T0* (*p* = 0.0110, *), T0 vs. T12 (*p* = 0.0087, **), and T0* vs. T12 (*p* = 0.0499, *) (Figure 9).

#### 3.4.5. Kinetic Data Analysis of Hypoglycin A Concentration

**Figure A3:**
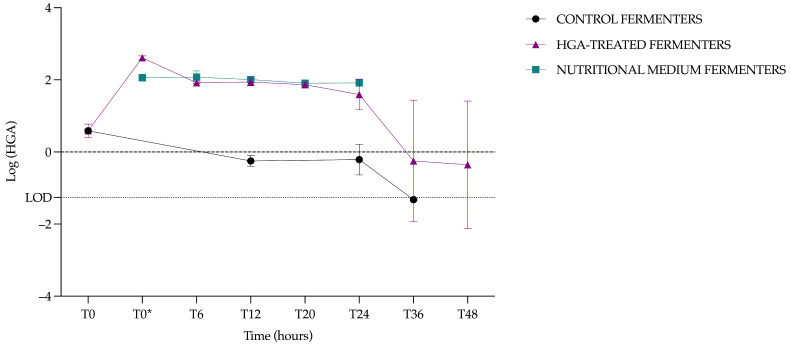
Graphical representation of log-transformed hypoglycin A (HGA) concentration in Nutritional Medium Fermenters, hypoglycin A-treated fermenters, and control fermenters.

The slope of the regression line was significantly steeper in the HTF (−3.80, *p* = 0.0156) compared with the CF (−0.09, *p* = 0.0011). No degradation occurred in the NMF (0.69, *p* = 0.6428). The linear regression analysis of HGA concentrations over time showed a significant decrease in both CF and HTF containing faecal microbiota, but not in the nutrient medium without microbiota (Figure A3, Appendix C).

### 3.5. Quantification of Methylenecyclopropylacetyl-Carnitine and Statistical Analysis

The results of MCPA-carnitine quantification in HTF were <LOD (i.e., 0.001 nmol/L) at T0, T2, T6, and T10; consequently, no further analyses were performed.

## 4. Discussion

Given the robust biochemical evidence linking HGA exposure to equine AM, the present study focuses specifically on HGA as the primary protoxin of interest, while recognising that co-exposure to MCPrG may occur in the natural setting

This study represents the first in vitro investigation of the equine colonic microbiota conducted under conditions that exclude any host–microbiota interactions, providing a controlled system to explore microbial mechanisms potentially relevant to equine AM. First, this model reveals differences in colonic bacterial populations between HGA-exposed and non-exposed conditions. Second, the study demonstrates that the protoxin HGA is specifically degraded by the descending colon microbiota of horses, as HGA remains stable in the sterile nutritive medium, without any detectable production of the toxic metabolite MCPA-carnitine.

The α-diversity analysis revealed significant differences between groups (i.e., CF vs. HTF) and within the HTF group. The reciprocal Simpson microbial diversity index was significantly impacted by the factor *Time* of the ANOVA analysis, which is consistent with the intrinsic functioning of the in vitro batch technique. Among the diversity indices, evenness exhibited significant differences between groups (i.e., CF vs. HTF), suggesting that HGA exposure mainly altered the relative distribution of taxa rather than community membership. At T12, the distribution of abundance (i.e., evenness) was significantly more uniform in the CF compared to the HTF, reflecting a more balanced representation of populations in the CF and suggesting that exposure to HGA may have selectively promoted or inhibited particular taxa early in the incubation. This pattern is consistent with in vivo observations, where horses classified as controls exhibited a more uniform distribution of microbial populations compared to co-grazers or intoxicated horses [32]. Moreover, the significant difference in evenness within HTF between T12 and T48 likely reflected the progressive disappearance of HGA, which alleviated selective pressures and allowed a more balanced distribution of taxa. Regarding the Figure A2, including T0 in the alpha diversity analysis, and as expected, T0 is similar between the groups. The decrease in diversity was also expected with the batch system, as well as the fact that this decrease is primarily due to a decrease in richness.

The *β*-diversity (i.e., between community) analysis, including T0, revealed that 66.8% of the microbial variation was significantly explained by the factor *Time*, the factor *Treatment* applied (i.e., adding HGA), and the interaction of both factors. Among these factors, *Time* exerted the strongest influence, consistent with the batch fermentation model where faecal inocula evolve in a closed environment mimicking part of the gastrointestinal tract. Moreover, the experimental design and the inclusion of T0 samples (i.e., representing a single baseline condition shared by all fermenters prior to any treatment) artificially inflated the explained variance and limited the ability of the permutation design to detect treatment-specific differences. When T0 samples were excluded, temporal variation remained the dominant factor structuring microbial communities. The PERMANOVA confirmed that *Time* alone significantly accounted for the largest share of variance (i.e., 26.2%), while the contribution of *Treatment* was comparatively small (i.e., 6.6%) and was not significant. This finding indicates that community changes over time were largely driven by intrinsic microbial succession rather than by direct HGA effects at specific sampling points. Consistent with these results, the Bray–Curtis distance-based RDA confirmed a strong and significant effect of Time on the first canonical axis, reinforcing the predominant role of temporal evolution in shaping the community structure.

To minimise the confounding influence of temporal dynamics and to better disentangle treatment-specific effects from the natural evolution of the microbial communities, DESeq2 analyses were performed by contrasting CF and HTF samples within each individual time point. The DESeq 2 analysis exploring CF vs. HTF at each time point (i.e., T12, T24, and T48) and for all times combined revealed that some bacterial populations are significantly different. Notably, the genus *Paraclostridium* was consistently more abundant in CF than in HTF samples at T12, T48, and when all times were combined. This genus is known to perform Stickland fermentation using leucine and BCAAs. The Stickland reaction is an anaerobic fermentation pathway in which one amino acid is oxidised as an electron donor, while another is reduced as an electron acceptor, thereby maintaining redox balance and generating ATP through substrate-level phosphorylation [62,63,64,65]. Hypoglycin A has a molecular weight close to that of BCAAs and can interfere with BCAA metabolic pathways [7]. Moreover, its structural similarity to leucine accounts for the chromatographic challenges in separating the two compounds [7]. Based on these properties, we hypothesise that *Paraclostridium* may have incorporated HGA instead of leucine or other BCAAs in Stickland fermentation, leading to an energy deficit and population collapse in the fermenters exposed to the toxin. To further validate these findings, the use of absolute quantification approaches, such as qPCR, would be valuable for determining whether the observed relative changes in bacterial populations genuinely reflect true increases or decreases in abundance. It is important to note that *Paraclostridium* was detected at a low relative abundance, suggesting that its direct impact on the overall community dynamics is likely limited. However, the Stickland reaction, a key metabolic pathway potentially involved in HGA utilisation, is not exclusive to *Paraclostridium* and can be carried out by other bacterial taxa as well. This raises the possibility that additional, more abundant populations could contribute substantially to the observed metabolic shifts in the fermenters. Consequently, a targeted investigation focusing on bacteria capable of performing Stickland fermentation could help identify the key taxa driving these functional responses and better elucidate the microbial mechanisms underlying HGA metabolism in this system. Such an approach would provide a more comprehensive understanding of both the compositional and functional impacts of the toxin and could guide future studies aimed at mitigating its effects in vivo. Interestingly, *Anaeroplasma* is present in CF but not in HTF, which is consistent with in vivo observation. Indeed, in a previous study, *Anaeroplasma* was significantly higher in horses considered as control horses vs. horses with AM [32]. Reductions in *Anaeroplasma* abundance have been reported in hypercholesterolemic subjects, a condition also characterised by altered lipid homeostasis [66]. Therefore, the observed depletion of *Anaeroplasma* in the HGA-treated batch may reflect the sensitivity of this genus to disruptions in fatty acid metabolism caused by the toxic metabolite MCPA-CoA.

The proportions of acetate, propionate, and butyrate are similar to those described in horses in the literature: 70–80%, 10–20%, and 5–10%, respectively, in the caecum and the colon [67]. Moreover, these concentrations of SCFAs indicate that the batch system worked as expected and without a significant difference between CF and HTF. The significant effect of *Time* observed for all three SCFAs reflects the natural progression of microbial fermentation in the batch model. The donor’s faeces were introduced into the system under conditions mimicking the colonic environment, and the microbiota were required to adapt to these conditions. Acetate appeared as early as T12 in all fermenters, whereas propionate and butyrate became detectable at T36 and T48, respectively—a pattern consistent with previous findings [35]. Propionate formation from pyruvate involves two major microbial pathways: the succinate pathway, which proceeds via oxaloacetate and succinate, and the acrylate pathway. These multi-step conversions likely contribute to the delayed accumulation of propionate in the system [67]. Butyrate is synthesised by strictly anaerobic bacteria and many of these anaerobic bacteria rely on acetate as a co-substrate or precursor via the butyryl-CoA/acetate CoA-transferase pathway. The delayed detection of butyrate and its higher fermenter-to-fermenter variability may reflect the complex microbial interactions and slower establishment of cross-feeding relationships required for its production [68,69,70]. Moreover, the multiple comparisons for each SCFA also revealed some interesting facts despite the presence of significant differences between CF and HTF. The acetate post hoc comparisons showed early significant differences in HTF compared to CF, which can be explained by the fact that HGA modifies the microbial composition associated with acetate production. The post hoc comparisons for propionate revealed a significant change only in HTF, suggesting the potential influence of HGA on metabolic pathways involved in propionate synthesis. Finally, the post hoc comparisons test for butyrate reflects the necessary time to produce this SCFA, but only in CF, suggesting a possible impact of HGA on butyrate-producing anaerobic bacteria. However, no significant difference was observed between groups (i.e., CF vs. HTF)

Hypoglycin A remained stable in the autoclaved nutritional medium at constant pH and temperature. In contrast, one study reported no change in HGA concentration after incubation with equine gastric or ovine rumen fluid for two hours [71]. However, this study did not consider the retention time in the sheep rumen, nor the fact that in horses, the majority of the gut microbiota is located in the colon rather than in the stomach. These methodological differences may explain why the results of that study do not align with our observations. Conversely, another study documented a decrease in HGA concentration in autoclaved ruminal fluid. Since sterilisation renders microorganisms non-viable, the authors attributed this reduction to abiotic processes, most likely related to changes in pH and/or temperature. This interpretation is consistent with the known stability of HGA in pure water [29] and supports our conclusion that, under certain conditions, HGA degradation can occur independently of active microbial metabolism. The chemical structure of HGA is composed of—as amino acids—one carboxyl group (-COOH) and one amine group (-NH_2_) linked to the same alpha carbon (Cα) and by a radical group composed of a methylene group (=CH_2_) adjacent to a cyclopropyl cycle composed of three carbon atoms. The carboxyl group and the amine group, which make HGA a hydrophilic molecule, can also influence its acid-base character. Indeed, the carboxyl group can release a proton (H^+^), making HGA a weak acid molecule. The pKa of the carboxyl group is generally between 2 and 3, as for most amino acids, indicating that it is dissociated (and therefore deprotonated) at physiological pH (~7.4). The amine group (-NH_2_) has a higher pKa, usually around 9–10, meaning it remains mostly protonated (NH_3_^+^) at physiological pH. Accordingly, at physiological pH, HGA exists primarily as a zwitterion, with the carboxyl group deprotonated (-COO^−^) and the amine group protonated (NH_3_^+^), giving it overall neutrality but with polar sites [72,73,74]. These polar sites could be potential sites for attracting other molecules. Consequently, the hypothesis of abiotic processes influencing the detection of HGA is a possibility. Especially since the concentration of HGA in our model of stability in an autoclaved medium with constant pH and temperature was stable during the experiment.

Within the CFs, the HGA concentrations drop to values close to or below LOD from T12 to T36. This significant decrease in HGA, in parallel with the stability in the nutritional medium, confirms that HGA is decreasing due to the activity of the microbiota in the batch system. In the HTFs, post hoc comparisons (Dunnett and Tukey) identified numerous significant differences between T0 (or T0*, immediately following HGA addition) and subsequent time points up to T20, indicating a progressive decline in HGA concentration as confirmed by the kinetic analysis. This trend, in parallel with the stability of HGA in the NMF, also suggests a microbial involvement.

In the HTFs, the kinetic profile of HGA degradation shows, at first, a decrease, followed by a plateau phase, and then a second decline. This biphasic pattern may reflect a mechanism of catabolite repression. Catabolite repression is a regulatory process in which microorganisms preferentially metabolise easily available carbon or nitrogen sources, repressing the expression of genes involved in the degradation of alternative or less favourable substrates [75]. In our system, the initial decrease in HGA may result from its direct utilisation by microorganisms, but the subsequent plateau suggests that once other nutrients in the medium are still readily available, microbial metabolism may shift away from HGA. The second decline observed after T20 could then indicate that, as preferred substrates become depleted, the microbial community resumes the degradation of HGA. This phenomenon is well described in microbial ecology and supports the idea that HGA degradation is not only possible but also subject to regulation depending on the nutritional context [75].

The two-way ANOVA comparing the CF and the HTF revealed significant effects of *Time* and *Treatment*. These findings collectively demonstrate a temporal evolution of HGA concentrations and a difference between the CF and HTF. As expected, HGA levels differed significantly between groups at T0*, T12, and T24, consistent with the timing of HGA exposure and subsequent microbial transformation. Moreover, HGA levels were not significantly different at T0 between CF and HTF (i.e., as no HGA had yet been added). Interestingly, results from a previous study on ruminal batch cultures provide a useful point comparison. In that system, the incubation of HGA in ruminal fluid batch cultures revealed a decrease in HGA over time, with a significant decrease after 8 h and undetectable levels after 24 h [29]. In the present study, within the HTF, the significant difference was already noted after 2 h and values <LOD after 48 h. Despite an initial concentration of HGA being relatively similar (i.e., 564 ± 133 ng/mL HGA in the sheep ruminal fluid and 411 ± 46 ng/mL HGA in the equine colonic fluid), the difference between the first time point of significant decrease in HGA (i.e., 8 h in sheep ruminal fluid and 2 h in equine colonic fluid) underlines the “*Time* × *Treatment*” effect observed. Moreover, this difference may be due to the difference in microbial composition between sheep ruminal fluid and horse colonic fluid. Indeed, the authors explained that the decrease in HGA in the active ruminal fluid was due to the activity of microbial enzymes in this medium via the integration of HGA (i.e., an amino acid) into microbial protein [29].

The kinetic analysis confirmed the results obtained by ANOVA, showing a clear decrease in HGA concentration over time only in the fermenters containing faecal microbiota. Moreover, the difference in the slope of regression in CF vs. HTF indicated a dose-dependent microbial degradation of HGA. The absence of a significant slope in NMF indicated that no degradation of HGA occurred in an autoclaved medium. As HGA concentrations remained unchanged in the NMF without microbiota during the initial incubation period, additional measurements at later time points were not performed.

The concordance between both approaches strengthens the evidence that HGA degradation occurs through microbial activity rather than abiotic processes.

The absence of MCPA-carnitine was expected as previously described in the literature on sheep ruminal fluid batch cultures (as well as MCPA-glycine) [29]. The absence of toxic metabolites in the sheep ruminal fluid (i.e., MCPA-carnitine, MCPA-glycine, MCPF-carnitine, and MCPF-glycine) [29] and in the present horse colonic batch system (i.e., MCPA-carnitine) demonstrates the absence of toxic metabolism (in the sense that conjugation with glycine and carnitine does not take place) of HGA (or MCPrG) in the digestive tract of herbivores. In the present study, HGA concentrations decreased markedly during the incubation in the faecal batch cultures, while remaining stable in the corresponding sterile nutritive medium, thereby confirming that its degradation was microbially mediated. Therefore, it can be assumed that if the digestive microbiota played a role in HGA poisoning, this role might be more “protective”, supporting the hypothesis that microbial activity promotes detoxification rather than activation into toxic metabolites. However, this detoxification occurs in the distal part of the digestive tract, suggesting that such microbial degradation may occur too late to prevent the host’s systemic uptake of the protoxin, which primarily happens in the small intestine. This interpretation is consistent with previous conclusions regarding proximal fermenter species, which stated that: “Their gut morphophysiology may act as a protection as toxins may be transformed in the rumen. Indeed, the degree of protection against A. pseudoplatanus may be directly linked to the rumen retention time of soluble molecules” [11]. These findings reinforce the hypothesis that the site and timing of microbial fermentation are critical determinants of susceptibility to HGA poisoning. Species in which fermentation occurs before the small intestine (e.g., ruminants) may inactivate HGA before absorption, whereas in hindgut fermenters like horses, the protoxin is likely absorbed upstream of microbial degradation. Moreover, this “protection” conferred by the proximal position of the fermentation tank could explain the greater number of HGA intoxication in horses compared to other herbivores, even considering differences in species management (i.e., the fact that horses are more often at pasture in autumn or spring compared to other herbivores as cattle or sheep)”. The implication of intestinal microbiota as a “protective actor” is already known for other phyto-protoxins as illustrated by mimosine metabolism in ruminants [76,77]. This example of mimosine metabolism in ruminants highlights that the ability of certain microbial communities to metabolise plant protoxins can evolve as an adaptive mechanism, conferring host tolerance to otherwise harmful compounds. Such examples illustrate the potential ecological and evolutionary relevance of microbial detoxification in herbivorous species. Despite its toxic effects, ruminants in certain regions tolerate mimosine ingestion due to microbial degradation of its goitrogenic and toxic metabolite [78,79]. Transfaunation experiments confirmed that ruminal microbiota confer resistance to mimosine toxicity [78,80]. Notably, *Synergistes jonesii*, an anaerobic bacterium isolated from Hawaiian goats, utilises the toxic metabolite for growth, demonstrating microbial adaptation to detoxification [81,82].

Gut microbiota is known to vary from one individual to another due to age or physiological status, but also due to several factors, such as diet, nutritional supplements, exercise, seasons, and medications [33]. To limit the individual impact on the faecal microbiota used in this study, two horses were selected, and faecal samples were pooled. Nevertheless, the number of horses in the present study could have been greater (n = 4) as suggested for in vitro fermentation studies [39]. Considering “age”, the two horses are considered adult horses and not mature or elderly horses; consequently, they did not exhibit a microbiome rearrangement observed in horses over 20 years of age [83] or a decline in bacterial *α* and *β*-diversity observed with advancing age [83,84]. The impact of sex is not clearly established in the literature, with contradictory results in horses [84,85]. Moreover, the horses involved in this study were not pregnant or lactating, cancelling out the possible impact of this specific physiological status on their intestinal microbiota [33]. Horses developing AM were at pasture for at least 6 h a day, and some of them were supplemented with hay [86,87]. Consequently, selected horses encountered this condition and received no grain or supplement: thus, their diet was a forage-based diet (i.e., grass and hay). Although differences exist in *α*-diversity of faecal microbiota between grass diet or grass/hay diet [88,89], these differences are less important compared to a diet partially composed of grains [90]. Fibrolytic bacteria are therefore expected, as these bacteria help break down fibre with the production of SCFAs [67,90]. The bacterial community in the forage-based diet is dominated by two phyla, Firmicutes and Bacteroidetes, which both account for more than 80% of the overall abundance of bacterial phyla [89]. Seasonal variation also influences faecal microbiota [84,89,91] either via the composition of the bacterial community in the environment (soil, grass, …) or via the nutrient composition of the pasture [89,92]. The main seasons at risk for AM are autumn and spring; horses involved in this study were sampled during spring 2022. Finally, the conservative procedure of storing faeces at −80 °C prior to the experimental procedure can be debated. Indeed, a recent study reported that freeze–thawing decreased the bacterial viability by 47% [93]. However, other studies have shown that storage at −80 °C does not lead to significant differences compared to fresh faecal samples, even over several months [94,95]. Consequently, some studies have used a temperature of −80 °C for the storage of their control samples for their analyses [96]. Moreover, other studies have demonstrated that storage at −20 °C does not induce significant changes in terms of relative abundance, α-diversity, and β-diversity [97,98].

The use of in vitro systems to study equine microbiota has already been scientifically approved and is considered reflective of in vivo conditions [35,36]. However, the absorption of amino acids, such as HGA (i.e., a small non-proteogenic amino acid [7,74]), mainly takes place in the jejunum and ileum, with a net disappearance of nitrogen (N) from 16% to 58% pre-caecally [47,99,100]. Consequently, the possibility that HGA reaches the colon in horses could be challenged by the distal location of this fermentation tank. However, studies about nitrogen dynamics in the equine gastrointestinal tract indicate that 11 to 30% of the apparent N digestion occurs in the small intestine vs. 40% to 70% in the hindgut [101,102,103]. Both cationic and neutral amino acid transporter genes are expressed in the equine large intestine, suggesting their potential role in microbial and dietary amino acid absorption [104]. In the caecal compartment, an injection of the 15N isotope of nitrogen led to the appearance of labelled essential and non-essential amino acids, urea, ammonia, and lysine in the caecal veins. These observations indicate that horses are able to digest and absorb microbial protein from the large intestine [105]. In humans, the microbiota is greater in the more distal part of the intestine compared to the proximal part of the intestine, where the concentrations of proteins, peptides, and amino acids are relatively high. Amino acids are not significantly absorbed by the colonic mucosa but are intensively metabolised by the large intestinal microbiota. The preferred amino acid substrates of human colonic bacteria include lysine, arginine, glycine, and the branched chain amino acids (i.e., leucine, valine, and isoleucine), resulting in the generation of a complex mixture of metabolic end products, including, among others, ammonia, SCFAs (acetate, propionate, and butyrate), and branched-chain fatty acids (valerate, isobutyrate, and isovalerate) [106].

Moreover, the unexpected presence of HGA in the faeces of the horses used in the present study (i.e., considered as control and toxin-free horses, since neither protoxin nor toxic metabolites were detected in their blood analysis) indicates that HGA does reach the distal part of the digestive tract and comes into contact with the colonic microbiota in vivo. This finding could be explained by the difference in sensitivity between the LOQ (i.e., 0.090 μmol/L) of the method used to quantify HGA in the blood of horses and the LOD (i.e., 0.055 ng/mL) of the method used to quantify HGA in the batched faecal samples and/or by the fact that part of the ingested amino acid is not absorbed in the small intestine and thus reaches the colon. Therefore, it can be hypothesised that the amino acids ingested by the horse such as HGA, may reach the caecal and colonic microbiota. Nevertheless, the presence of HGA in the faeces of horses selected as “toxin-free” may have already influenced the composition of their colonic microbiota, potentially introducing a bias in the present experimental design.

Future research should aim to fully unravel the fate of HGA within the gut ecosystem and its interactions with microbial communities. Radiolabelled carbon tracing could provide decisive insights into the metabolic pathways involved and clarify whether HGA is transformed, assimilated, or detoxified by specific microbial taxa. In parallel, metabolomic approaches could comprehensively profile the chemical transformations of HGA and related metabolites, allowing a deeper understanding of how microbial activity shapes the local metabolic landscape. Beyond mechanistic understanding, the identification of bacterial groups with protective potential would pave the way for innovative interventions, ranging from microbial transfaunation strategies to the rational design of next-generation probiotics. Such approaches hold the promise not only of mitigating the risks associated with HGA exposure in equids but also of advancing our broader comprehension of host–microbiota interactions in the context of xenobiotic metabolism.

## 5. Conclusions

For the first time, an in vitro experiment confirmed that the equine intestinal microbiota is modified by the presence of HGA. Moreover, the decrease in the protoxin concentration during the experiment supports the protective role of the microbiota, given that HGA remains stable in autoclaved nutritive media without faecal inocula. Similar observations have also been reported previously in sheep ruminal content. In particular, one genus, *Paraclostridium*, was identified as having a significant impact on the system. Further investigations are needed to confirm which genera or species are involved in HGA degradation. Understanding which microorganisms participate in this process could pave the way for potential therapeutic strategies.

## Figures and Tables

**Table 1:** *β*-diversity pairwise comparisons without T0.

Pairs	*p*-Value Adjusted	
T12 vs. T24	0.5416	nsig
T12 vs. T48	0.0189	*
T24 vs. T48	0.0265	*

Significantly different with a *p*-value of 0.05 or less: * <0.05; nsig: not significant; CF: control fermenters; HTF: hypoglycin A-treated fermenters.

## Data Availability

Raw amplicon sequencing libraries were submitted to the NCBI database under bioproject number PRJNA1335877.

## Abbreviations

The following abbreviations are used in this manuscript:

AM	Atypical myopathy
HGA	Hypoglycin A
MCPrG	Methylenecyclopropylglycine
BCAA	Branched-chain amino acids
MCPA-CoA	Methylenecyclopropylacetyl-coenzyme A
MCPA-carnitine	Methylenecyclopropylacetic acid-carnitine
MCPA-glycine	Methylenecyclopropylacetic acid-glycine
UPLC-MS/MS	Ultra-performance liquid chromatography with mass spectrometry
LOQ	Limit of quantification
LOD	Limit of detection
MTD	Maximum tolerated dose
HED	Human equivalent dose
BSA	Body surface area
CFs	Control fermenters
HTFs	HGA-treated fermenters
SCFAs	Short-chain fatty acids
PCR	Polymerase chain reaction
ANOVA	Analysis of variance
PERMANOVA	Permutational multivariate analysis of variance
dbRDA	Distance-based redundancy analysis
SPME	Solid phase microextraction
GC-MS	Gas chromatography coupled to mass spectrometry
LLOQ	Lower limits of quantification
ULOQ	Upper limits of quantification
NMF	Nutritional medium fermenters
FDR	False discovery rate
